# Machine learning models for predicting postoperative peritoneal metastasis after hepatocellular carcinoma rupture: a multicenter cohort study in China

**DOI:** 10.1093/oncolo/oyae341

**Published:** 2025-01-20

**Authors:** Feng Xia, Qian Chen, Zhicheng Liu, Qiao Zhang, Bin Guo, Feimu Fan, Zhiyuan Huang, Jun Zheng, Hengyi Gao, Guobing Xia, Li Ren, Hongliang Mei, Xiaoping Chen, Qi Cheng, Bixiang Zhang, Peng Zhu

**Affiliations:** Department of Hepatic Surgery, Tongji Hospital, Tongji Medical College of Huazhong University of Science and Technology, Wuhan, Hubei, People’s Republic of China; Department of Hepatic Surgery, Tongji Hospital, Tongji Medical College of Huazhong University of Science and Technology, Wuhan, Hubei, People’s Republic of China; Department of Hepatic Surgery, Tongji Hospital, Tongji Medical College of Huazhong University of Science and Technology, Wuhan, Hubei, People’s Republic of China; Department of Emergency Medicine, Zhongshan People’s Hospital Affiliated to Guangdong Medical University, Zhongshan, Guangdong, People’s Republic of China; Department of Hepatic Surgery, Tongji Hospital, Tongji Medical College of Huazhong University of Science and Technology, Wuhan, Hubei, People’s Republic of China; Department of Hepatic Surgery, Tongji Hospital, Tongji Medical College of Huazhong University of Science and Technology, Wuhan, Hubei, People’s Republic of China; Department of Hepatic Surgery, Tongji Hospital, Tongji Medical College of Huazhong University of Science and Technology, Wuhan, Hubei, People’s Republic of China; Department of Science and Education, Shenzhen Baoan District People’s Hospital, Shenzhen, Guangdong, People’s Republic of China; Department of Hepatobiliary and Pancreatic Surgery, Shenzhen Longhua District People’s Hospital, Shenzhen, Guangdong, People’s Republic of China; Department of Hepatobiliary and Pancreatic Surgery, Huangshi Central Hospital, Hubei Polytechnic University, Huangshi, Hubei, People’s Republic of China; Department of Hepatobiliary Surgery, Affiliated Hospital of Qinghai University, Xining, Qinghai, People’s Republic of China; Department of General Surgery, General Hospital of Central Theater Command, Wuhan, Hubei, People’s Republic of China; Department of Hepatic Surgery, Tongji Hospital, Tongji Medical College of Huazhong University of Science and Technology, Wuhan, Hubei, People’s Republic of China; Department of Hepatic Surgery, Tongji Hospital, Tongji Medical College of Huazhong University of Science and Technology, Wuhan, Hubei, People’s Republic of China; Department of Hepatic Surgery, Tongji Hospital, Tongji Medical College of Huazhong University of Science and Technology, Wuhan, Hubei, People’s Republic of China; Department of Hepatic Surgery, Tongji Hospital, Tongji Medical College of Huazhong University of Science and Technology, Wuhan, Hubei, People’s Republic of China

**Keywords:** rupture hepatocellular carcinoma, peritoneum, metastasis, prognostic factors, machine learning

## Abstract

**Background:**

Peritoneal metastasis (PM) after the rupture of hepatocellular carcinoma (HCC) is a critical issue that negatively affects patient prognosis. Machine learning models have shown great potential in predicting clinical outcomes; however, the optimal model for this specific problem remains unclear.

**Methods:**

Clinical data were collected and analyzed from 522 patients with ruptured HCC who underwent surgery at 7 different medical centers. Patients were assigned to the training, validation, and test groups in a random manner, with a distribution ratio of 7:1.5:1.5. Overall, 78 (14.9%) patients experienced postoperative PM. Five different types of models, including logistic regression, support vector machines, classification trees, random forests, and deep learning (DL) models, were trained using these data and evaluated based on their receiver operating characteristic curve and area under the curve (AUC) values and F1 scores.

**Results:**

The DL models achieved the highest AUC values (10-fold training cohort: 0.943, validation set: 0.928, and test set: 0.892) and F1 scores (10-fold training set: 0.917, validation cohort: 0.908, and test set:0.899) The results of the analysis indicate that tumor size, timing of hepatectomy, alpha-fetoprotein levels, and microvascular invasion are the most important predictive factors closely associated with the incidence of postoperative PM.

**Conclusion:**

The DL model outperformed all other machine learning models in predicting postoperative PM after the rupture of HCC based on clinical data. This model provides valuable information for clinicians to formulate individualized treatment plans that can improve patient outcomes.

**Implications for practice**

Early identification of high-risk patients: The development of machine learning models, particularly the deep learning model, provides a robust tool for predicting the risk of PM after rHCC surgery. Clinicians can utilize these models to identify high-risk patients early, allowing for targeted interventions aimed at preventing the occurrence of PM. This will enable better individualized treatment planning and closer monitoring of at-risk patients to improve prognosis. Timing of hepatectomy: The study underscores the importance of early staged hepatectomy (SEPH) in reducing the incidence of postoperative PM. Clinicians are encouraged to prioritize SEPH when possible, as this approach has been associated with a lower risk of metastasis and better patient outcomes. Surgical timing should be carefully evaluated, particularly in cases where SEPH is feasible, to optimize the chances of reducing metastasis. Integration of predictive models into clinical workflow: The findings support the integration of machine learning models into the clinical decision-making process. By incorporating patient-specific data into these models, healthcare providers can assess postoperative risks more accurately and make informed decisions about the frequency of follow-up imaging and the need for early interventions, such as chemotherapy or targeted therapies, to address micrometastases. Intraoperative measures: For patients predicted to be at high risk of PM, surgeons can implement intraoperative measures, such as peritoneal lavage with sterile water, to reduce the likelihood of peritoneal implantation of free cancer cells. This approach may help improve long-term outcomes for patients undergoing surgery for ruptured HCC. Personalized postoperative monitoring and treatment: Patients identified as high-risk for PM through these predictive models should be subjected to more frequent imaging follow-ups, such as every 3 months, to detect metastases early. In cases where early metastases are identified, more aggressive postoperative management, including systemic therapies, can be initiated promptly.

## Introduction

Hepatocellular carcinoma (HCC) rupture is a life-threatening complication with a notable incidence in some parts of Asia. Additionally, male patients, those with larger tumors, and patients with advanced liver disease are at higher risk of HCC rupture.^1^ Patients with ruptured liver cancer usually present with acute abdominal pain, hypotension, and shock, symptoms caused by massive intraperitoneal hemorrhage resulting from the tumor rupture. In some cases, patients may also exhibit hepatic tenderness and upper abdominal masses.^2^ The exact pathological mechanisms of HCC rupture have not been fully elucidated but may be related to the following factors: increased tension in the liver capsule caused by the large tumor size, increased vascular fragility, and vascular rupture triggered by external trauma or hypertension. Tumor necrosis, hemorrhage, and rapid growth within the tumor may further predispose it to rupture.^1^ Rupture of HCC has been considered a highly fatal complication with an acute phase mortality rate of 25%-75% if effective interventions are not taken.^1^ However, staged curative surgery is currently recognized as a treatment method for ruptured hepatocellular carcinoma (rHCC).^3^ In some early stage rHCC patients, surgical intervention can achieve a prognosis similar to that of non-ruptured HCC patients.^4^ Nevertheless, multiple studies have shown that intra-abdominal implantation metastasis of liver cancer, especially after HCC rupture, often occurs, and postoperative abdominal cavity implantation metastasis has a significant impact on the prognosis of rHCC patients.^5,6^ The appearance of peritoneal implantation metastasis after rHCC surgery will result in worse survival. Therefore, it is crucial to prevent such negative outcomes from occurring.

The cause of intra-abdominal metastasis is generally believed to be the preoperative presence of free cancer cells in the abdominal cavity, especially in ruptured HCC. These cancer cells are considered as some tumor-active “seeds” that have spread stealthily in the peritoneal cavity.^7^ At the same time, surgery-induced cancer cell dissemination and decreased immune function promote the implantation and growth of free cancer cells, especially beneath the liver, since the surrounding tissues of the liver often adhere to tumor cells after forming adhesions with the tumor.^8-10^ During liver cancer resection, contamination is more likely to occur around the liver. For rHCC patients who develop PM, their postoperative prognosis will significantly deteriorate. Roussel et al^6^, using multicenter data from Europe, observed that the OS rate for rHCC patients with peritoneal metastasis (PM) was inferior to that of single-lesion intrahepatic recurrence patients, but the risk factors for PM were not clear. In addition, Asian researchers have also shown that the occurrence of PM is associated with poorer prognosis.^11,12^ Therefore, predicting postoperative PM after rHCC surgery is crucial, and efforts should be made to prevent its occurrence. Early interventions should be performed for high-risk patients of postoperative PM to prolong their survival.

Therefore, we used a multicenter prospective maintained dataset to construct various machine learning models for predicting the occurrence of postoperative PM and compared these models. By employing multiple machine learning models, clinicians can intervene early in high-risk rHCC patients and improve their prognosis. We present this article in accordance with the STROBE reporting checklist.

## Materials and methods

### Patient selection

We retrospectively analyzed 522 rHCC patients who underwent radical hepatectomy in a prospectively maintained database collected on a continuous basis at 7 centers between December 2018 and December 2021. Postoperative peritoneal implantation metastatic in patients with ruptured liver cancer refers to the spread of free cancer cells from the primary site into the peritoneal cavity after ruptured liver cancer surgery and the formation of new tumor nodules or metastases by growing on the peritoneal surface or abdominal organs through implantation, with the metastases confirmed by 2 experienced imaging specialists together. In routine postoperative follow-up, the detection of nodules or omental nodules on the surface of abdominal organs and the presence of intraperitoneal fluid on computed tomography (CT) and magnetic resonance imaging (MRI) suggest PM. R0 margin is a condition where no residual cancerous tissue is seen on pathological examination, and an R1 margin is a condition where residual cancerous tissue is found to be present within the surgical margin on pathological examination. Postoperative pathology was issued jointly by 2 experienced pathologists. All patients were subjected to strict inclusion and exclusion criteria, inclusion criteria were (1) HCC confirmed by pathologists, (2) 2 preoperative imaging findings suggestive of tumor rupture, (3) R0 resection, and (4) no prior history of HCC or other malignancies; exclusion criteria were (1) previous antitumor therapy, (2) combination of other types of tumors, and (3) incomplete clinical data. This study was approved by the Ethics Committees of Wuhan Tongji Hospital, Zhongshan People’s Hospital, Huangshi Central Hospital, Shenzhen Baoan District People’s Hospital, Shenzhen Longhua District People’s Hospital, Affiliated Hospital of Qinghai University, and General Hospital of Central Theater Command, and informed consent was obtained from all patients. This study also adhered to the Strengthening the Reporting of Observational Studies in Epidemiology (STROBE) statement.

### Treatment approach

Seven medical centers adopted a unified and standardized treatment strategy. All patients with rHCC were admitted to the Intensive Care Unit for close monitoring, and those in the acute phase received appropriate treatment to maintain hemodynamic stability, including fluid resuscitation and blood transfusion if necessary. Transarterial embolization/ chemoembolization and conservative therapy were the main treatment options for hemodynamic instability caused by acute abdominal bleeding. For patients with preserved liver function in whom bleeding could not be controlled or was ineffective, emergency surgery was performed. The aim of emergency surgery was to perform curative intent resection on resectable tumors while simultaneously achieving hemostasis. For resectable rHCC patients who did not require emergency surgery, staged delayed hepatectomy was performed after a detailed professional evaluation upon admission, provided that bleeding had stopped and hemodynamics were stable after initial treatment.

Based on prior studies conducted at our Center, 2 distinct approaches were established for partial hepatectomy after HCC rupture: staged early partial hepatectomy (SEPH), performed within ≤8 days, and staged delayed partial hepatectomy (SDPH), performed more than 8 days post HCC rupture.^13^ Preoperative assessment included imaging (abdominal ultrasound, enhanced CT, and abdominal MRI), cardiopulmonary and renal evaluation, serology, Model for End-Stage Liver Disease score, Child-Pugh score, albumin-bilirubin score, 3D liver reconstruction, etc. All rHCC patients who underwent hepatectomy received curative intent resection. During the process of liver segment resection, the Pringle maneuver was used for hepatic inflow occlusion if necessary. All patients underwent multi-disciplinary treatment before surgery to determine the optimal surgical approach and postoperative management.

### Data collection and feature selection

The patients’ demographic characteristics, preoperative laboratory findings, and surgical details were collected and analyzed. After data collection, all 22 variables were included in the model, and feature selection was performed using univariate analyses to screen for potential predictors with a *P*-value less than.05. Variables were then included in a multivariate logistic regression model, and recursive feature elimination was applied to further select the most informative features, using a random forest (RF) algorithm to calculate feature importance scores (Table 1).

**Table 1.** Baseline characteristics of patients with rHCC undergoing resection in the training, validation, and test cohorts (*n* = 522).

	Training cohort (*n* = 366)	Validation cohort (*n* = 78)	Test cohort (*n* = 78)	*P*-value^b^
Gender				.649
Male	309 (84.4)	67 (85.9)	69 (88.5)	
Female	57 (15.6)	11 (14.1)	9 (11.5)	
Age (years)				.324
<60	322 (88.0)	64 (82.1)	66 (84.6)	
≥60	44 (12.0)	14 (17.9)	12 (15.4)	
Tumor max length				.654
≤8 cm	267 (73.0)	55 (70.5)	60 (76.9)	
>8 cm	99 (27.0)	23 (29.5)	18 (23.1)	
Tumor number				.702
Single	275 (75.1)	60 (76.9)	62 (79.5)	
Multiple	91 (24.9)	18 (23.1)	16 (20.5)	
AFP				.966
≤400 ng/mL	177 (48.3)	39 (50.0)	38 (48.7)	
>400 ng/mL	189 (51.7)	39 (50.0)	40 (51.3)	
Cirrhosis				.576
No	283 (77.3)	59 (75.6)	56 (71.8)	
Yes	83 (22.7)	19 (24.4)	22 (28.2)	
Differentiation grade				.534
Edmondson-Steiner I/II	224 (61.2)	49 (62.8)	53 (67.9)	
Edmondson-Steiner III/IV	142 (38.8)	29 (37.2)	25 (32.1)	
Child-Pugh				.410
A	295 (80.6)	58 (74.4)	60 (76.9)	
B	71 (19.4)	20 (25.6)	18 (23.1)	
BCLC stage				.174
A	200 (54.6)	49 (62.8)	50 (64.1)	
B	166 (45.4)	29 (37.2)	28 (35.9)	
MVI				.681
No	217 (59.3)	42 (61.8)	44 (64.7)	
Yes	149 (40.7)	26 (38.2)	24 (35.3)	
Satellite foci				.759
No	229 (62.6)	48 (61.5)	52 (66.7)	
Yes	137 (37.4)	30 (38.5)	26 (33.3)	
HBsAg				.971
No	50 (13.7)	11 (14.1)	10 (12.8)	
Yes	316 (86.3)	67 (85.9)	68 (87.2)	
ALB				.943
≤35 g/L	166 (45.4)	37 (47.4)	36 (46.2)	
>35 g/L	200 (54.6)	41 (52.6)	42 (53.8)	
ALT				.512
≤50 U/L	291 (79.5)	58 (74.4)	59 (75.6)	
>50 U/L	75 (20.5)	20 (25.6)	19 (24.4)	
AST				.136
≤40 U/L	208 (56.8)	38 (48.7)	36 (46.2)	
>40 U/L	158 (43.2)	40 (51.3)	42 (53.8)	
ALP				.527
≤100 U/L	288 (78.7)	62 (79.5)	74 (84.1)	
>100 U/L	78 (21.3)	16 (20.5)	14 (15.9)	
GGT				.811
≤60 U/L	211 (57.7)	47 (60.3)	43 (55.1)	
>60 U/L	155 (42.3)	31 (39.7)	35 (44.9)	
Perioperative factors				
Blood loss (mL)^a^	350 [150-1200]	370 [200-1300]	420 [140-1250]	.316^c^
Perioperative blood transfusion				.807
No	220 (60.1)	45 (57.7)	49 (62.8)	
Yes	146 (39.9)	33 (42.3)	29 (37.2)	
Timing of hepatectomy				.995
SEPH	241 (65.8)	51 (65.4)	51 (65.4)	
SDPH	125 (34.2)	27 (34.6)	27 (34.6)	
Hepatectomy time				.569
≤220 minutes	194 (53.0)	46 (59.0)	40 (51.3)	
>220 minutes	172 (47.0)	32 (41.0)	38 (48.7)	
Extent of hepatectomy				.703
Major hepatectomy	188 (51.4)	39 (50.0)	36 (46.2)	
Minor hepatectomy	178 (48.6)	39 (50.0)	42 (53.8)	
Time of inflow occlusion (minutes)	14 [9-18]	13 [8-17]	14 [9-19]	
Postoperative PM				.311
No	314 (85.8)	68 (87.2)	62 (79.5)	
Yes	52 (14.2)	10 (12.8)	16 (20.5)	

^a^Median (range).

^b^χ^2^ test with Yates’ correction.

^c^Wilcoxon rank-sum test.

The values in parentheses are percentages unless indicated otherwise.

Abbreviations: AFP, alpha-fetoprotein; ALBI, albumin-bilirubin grade; ALP, alkaline phosphatase; ALT, alanine aminotransferase; AST, aspartate aminotransferase; BCLC, Barcelona Clinic Liver Cancer; GGT, γ-glutamyl transpeptidase; HBsAg, hepatitis B surface antigen; HCC, hepatocellular carcinoma; MVI, microvascular invasion; PM, peritoneal metastasis; rHCC, ruptured hepatocellular carcinoma; SEPH, staged early partial hepatectomy; SDPH, staged delayed partial hepatectomy; TACE, transcatheter arterial chemoembolization.

### Machine learning models development

After the feature selection process, 4 different machine learning models were developed and trained to predict the risk of PM after hepatic rupture in HCC patients: classification tree model (CTM), RF, support vector machine (SVM), and a fully connected neural network model. The classification tree recursively splits the dataset to create a decision tree for classifying new data points. RF is an ensemble method that combines multiple decision trees for improved accuracy. SVM finds the hyperplane that maximally separates classes, while a fully connected neural network model utilizes interconnected layers of neurons to learn complex relationships between inputs and outputs.

The fully connected deep neural network model used 2 hidden layers, comprising 128 and 64 neurons, respectively. All layers, except the final one, used ReLU activation functions, while the last layer used a sigmoid activation function.

For the CTM, we used the CART algorithm with default hyperparameters, including a maximum depth of 3 and a maximum number of 10 leaf nodes. These hyperparameters control the complexity and size of the resulting decision tree. The maximum depth restricts the number of levels or layers in the tree, limiting its overall complexity and preventing overfitting. The maximum number of leaf nodes specifies the maximum number of terminal nodes or leaves in the tree, allowing for a more compact representation of the model while still capturing important patterns in the data. For the RF model, we used 500 decision trees with a maximum depth of 10 and a minimum number of samples required to split each node set to 2. For the SVM model, we used a radial basis function (RBF) kernel with C = 1 and gamma = 0.1.

### Model evaluation

We divided the entire dataset into a training set (70%), a validation set (15%), and a test set (15%). The training set was used for model training and cross-validation, the validation set was used for hyperparameter tuning, and the test set was used to evaluate the model’s final generalization capability. The test set data were completely independent of the model’s training and tuning processes. To enhance the model’s robustness and reduce bias from a single data split, we performed 10-fold cross-validation on the training set. Specifically, the training set was randomly divided into 10 non-overlapping subsets, with 9 subsets used for training and the remaining 1 subset used for validation in each iteration. This process was repeated 10 times to ensure that each subset served as a validation set once. We calculated performance metrics (such as receiver operating characteristic [ROC]-area under the curve [AUC], accuracy, recall, F1 score, etc.) for each fold and reported the average performance and standard deviation for each model. After completing the 10-fold cross-validation, we used the validation set for hyperparameter tuning. By adjusting parameters such as regularization strength and learning rate, we selected the model configuration that performed best on the validation set. Once hyperparameter tuning was finished, we conducted an independent evaluation of the final model using the test set. The test set data were never involved in the model’s training or validation and were used to assess the model’s generalization ability. We reported the performance metrics of the model on the test set, including ROC-AUC, accuracy, recall, and F1 score.

We evaluated the models with several performance metrics, including accuracy, AUC-ROC, sensitivity, specificity, positive predictive value, and negative predictive value. We also performed a grid search to find the optimal hyperparameters for each model. For the classification tree and RF models, we varied the maximum depth and the minimum number of samples required to segment each node. For the SVM model, we changed the C and gamma parameters. For the deep neural network model, we changed the number of neurons in each hidden layer.

The best-performing model was selected based on its AUC-ROC score and F1 score.

### Follow-up

Patients were reviewed with imaging in the first month after discharge, followed by follow-up every 3 months for the first year and every 6 months thereafter, with imaging (eg, enhanced CT and abdominal MRI) and laboratory tests (including liver and kidney function tests, electrolytes, and tumor markers) performed at each follow-up visit. OS was defined as the time from the date of surgery to death. Follow-up continued until May 30, 2023.

### Data analysis

Continuous variables were presented as median with their respective ranges, while categorical variables were expressed as frequencies with percentages. For comparing categorical variables, the chi-square or Fisher’s exact test was used. Survival rates for OS were analyzed using Kaplan–Meier curves with the log-rank test. To evaluate factors influencing OS after hepatectomy, both univariate and multivariate Cox regression analyses were performed. In the multivariate regression analysis, only variables with a significance level of *P* < .05 from the univariate analysis were included. The backward stepwise approach was used for variable entry. An automated iteration number selection method was implemented to ensure model convergence and smooth fitting. To mitigate treatment selection bias and other potential confounding factors, an inverse probability treatment weighting (IPTW) analysis was conducted. Propensity scores were calculated through a logistic regression model that incorporated several covariates, including age, sex, serum total bilirubin level, Child-Pugh class, background liver disease, tumor-node-metastasis (TNM) stage, tumor size, and tumor number.

Statistical analyses were executed using SPSS 25.0 software, with *P*-values < .05 (2-sided) considered statistically significant. Python (version 3.7.6; Python Software Foundation) was used for machine learning model building. Sample size estimation was performed using PASS software (version: 11.0). Additionally, R software was used to generate Kaplan–Meier curves, ROC curves, and PR curves.

## Results

### Baseline information table for rHCC patients in the training group, validation group, and test group

**Figure 1.**
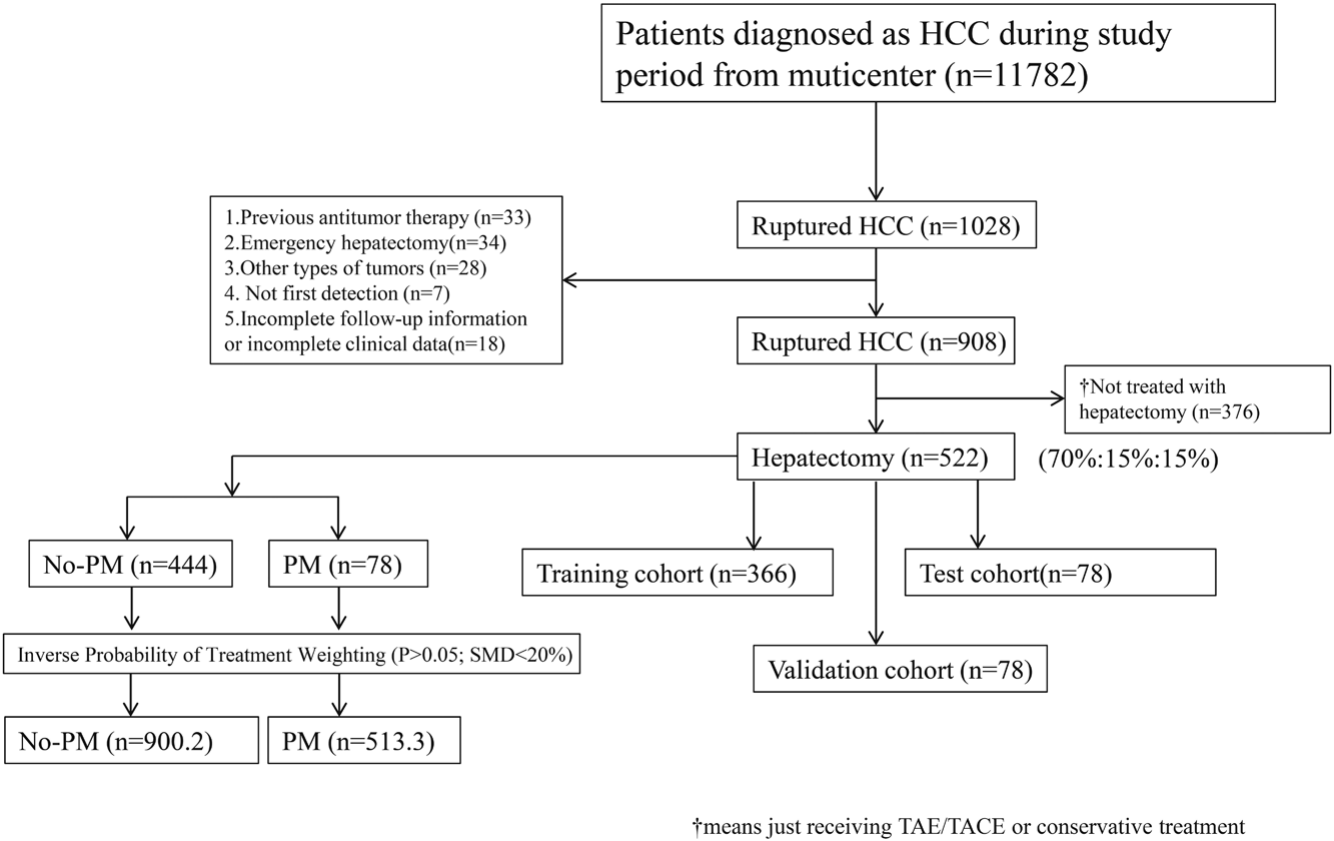
Flow chart for inclusion and exclusion of rHCC patients in 7 centers.

A retrospective collection was conducted on 522 patients who underwent radical surgery at 7 central hospitals. The patients were randomly allocated into training, validation, and test cohorts using a 7:1.5:1.5 ratio, comprising 366 patients in the training cohort, 78 patients in the validation cohort, and 78 patients in the test cohort. The inclusion and exclusion process of the patients is visually represented in Figure 1. In the training cohort, there were 309 male patients, accounting for 84.4% of the total. Among the patients, 99 had tumors with a max length greater than 8 cm, representing 27.0%. The majority of patients (275 individuals) had solitary tumors. Before surgery, the liver function of most patients was graded as Child-Pugh A, with 295 patients, constituting 80.6% of the cohort. Furthermore, a majority of patients underwent SEPH, with 241 patients, making up 65.8%. No statistically significant differences (*P* > .05) were observed in all variables between the 3 groups. Detailed data for both patient groups are presented in Table 1.

### Comparison of baseline characteristics between the PM and no-PM groups in all rHCC patients before and after IPTW

Baseline characteristics comparison before IPTW revealed that among 522 patients, 444 (85.1%) had no postoperative PM (No-PM group) and 78 (14.9%) experienced postoperative PM (PM group). In the No-PM group, there were 380 males (85.6%) and 64 females (14.4%), with 58 patients (13.1%) aged 60 years or older and 386 patients (86.9%) aged below 60 years. In the PM group, there were 65 males (83.3%) and 13 females (16.7%), with 12 patients (15.4%) aged 60 years or older and 66 patients (84.6%) aged below 60 years. The 3 groups exhibited imbalance in variables such as gender, Barcelona Clinic Liver Cancer (BCLC) stage, alpha-fetoprotein (AFP), cirrhosis, differentiation grade, microvascular invasion (MVI), satellite foci, hepatitis B surface antigen, timing of hepatectomy, perioperative blood transfusion, times of hilar inflow occlusion (HIO), and time of inflow occlusion. The PM group had a higher frequency of blood loss and perioperative blood transfusion, as well as a significantly higher proportion of delayed liver resection in advanced stages. After IPTW, the baseline characteristics of the 3 groups were balanced (Table S1). See Figure S1 for standardized mean difference (SMD) changes before and after IPTW matching. Before IPTW, the OS rates of the No-PM group at 1-, 3-, and 5-year were 66.4%, 36.6%, and 25.8%, respectively, while in the PM group, the rates were 51.3%, 15.4%, and 11.5%, respectively. There was a significant statistical difference in survival between the 2 groups (*P* < .05). After IPTW, there remained a statistically significant difference in OS rates between the 2 groups (*P* < 0.05) (Figure S2). Multivariate COX regression analysis suggested BCLC staging (hazard ratio [HR] = 1.823 [1.389-2.397], *P* < .001, AFP (HR = 1.655 [1.312-2.078], *P* < .001), tumor max length (HR = 1.655 [1.312-2.078], *P* < .001), differentiation grade (HR = 1.590 [1.278-1.979], *P* < .001), MVI (HR = 1.701 [1.304-2.219], *P* < .001), s foci (HR = 2.259 [1.788-2.852], *P* < .001), AST (HR = 1.466 [1.121-1.916], *P* = .005), ALP (HR = 1.670 [1.207-2.310], *P* = .002), GGT (HR = 1.351 [1.028-1.776], *P* = .031), timing of hepatectomy (HR = 2.081 [1.681-2.713], *P* < .001), extent of hepatectomy (HR = 1.778 [1.317-2.556], *P* < .001), and PM (HR = 1.388 [1.179-1.988], *P* < .001) (Table S2). The comparison of postoperative complications between the 2 groups did not show any statistically significant difference, and only Distant metastasis of liver cancer was statistically different (*P* < .001) (Table S3).

### Model characteristics of different predictors of postoperative PM and the ROC curve

**Figure 2.**
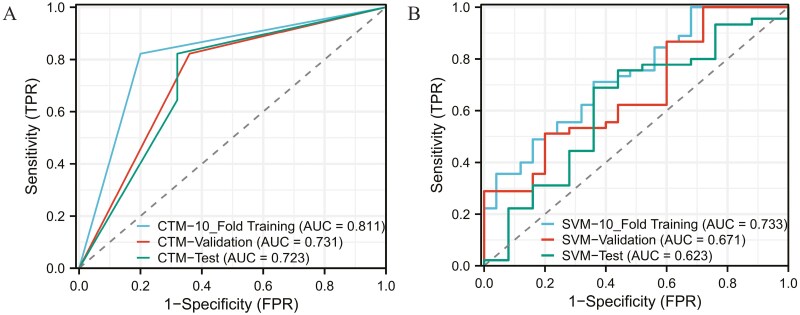
A represents ROC curves of the classification tree model in the 10-fold training, validation, and test cohorts; B represents ROC curves of the SVM model in the 10-fold training, validation, and test cohorts.

**Figure 3.**
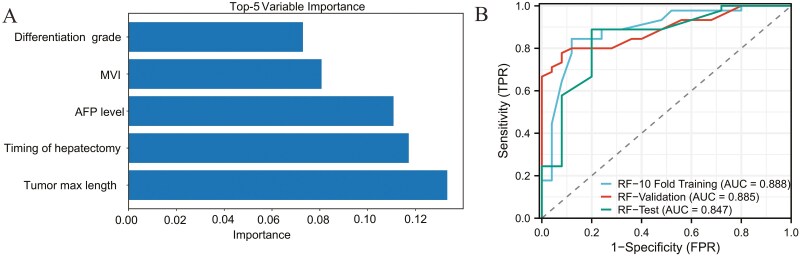
Evaluation of the random forest model. A represents the 5 most important variables in the model, and B represents the ROC curves of the random forest model in the 10-fold training, validation, and test cohorts.

**Figure 4.**
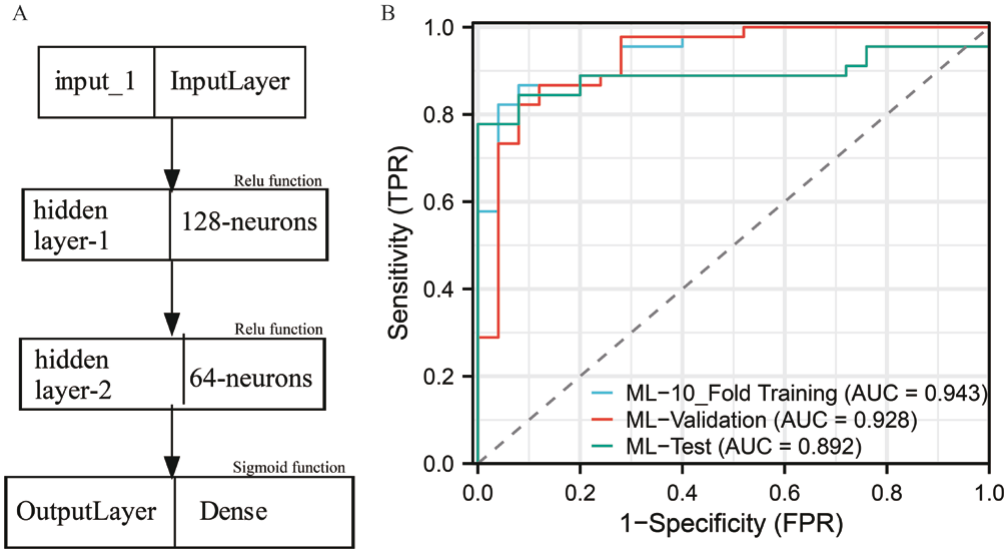
Evaluation of the deep learning model. A represents the basic framework for deep learning models, and B represents the ROC curves of the DL model in the 10-fold training, validation, and test cohorts.

Based on the results of the multivariable logistic regression analysis, a logistic model was constructed (Table 2). The area under the ROC curve for the 10-fold training, validation, and test sets were 0.915, 0.908, and 0.891, respectively (Figure S3). After multiple parameter adjustments, the decision tree model was optimized with a maximum tree depth of 3, a maximum number of leaf nodes of 10, and the Gini index as the splitting criterion. The final decision tree is depicted in Figure S4, with the area under the ROC curve of 0.811 for the 10-fold training set, 0.731 for the validation set, and 0.723 for the test set (Figure 2A). In the case of the SVM model, an RBF kernel with C = 1 and gamma = 0.1 was used, resulting in AUC values of 0.733 for the 10-fold training cohort, 0.671 for the validation cohort, and 0.623 for the test cohort (Figure 2B). Similarly, after multiple parameter adjustments in the RF model, 500 decision trees were used with a maximum depth of 10, and the minimum number of samples required for each split was set to 2. The top 5 most important variables in the model were identified as tumor max length, timing of hepatectomy, AFP level, MVI, and differentiation grade (Figure 3A). The ROC curve analysis demonstrated an area under the curve (AUC) of 0.888 for the 10-fold training cohort, 0.885 for the validation cohort, and 0.847 for the test cohort (Figure 3B). Finally, for the deep neural network model, 2 hidden layers with 128 and 64 neurons, respectively, were used. Except for the last layer, which used a sigmoid activation function, all other layers used the ReLU activation function. The architecture of the model is presented in Figure 4A, and the AUC of the ROC curve for the 10-fold training, validation, and test sets was 0.943, 0.928, and 0.892, respectively (Figure 4B).

**Table 2.** Univariate and multivariate logistic regression analyses of risk factors associated with PM following hepatectomy for rHCC in the training cohort.

			Univariate analysis			Multivariate analysis		
Variables	Number	Percent	*P*	OR	95% CI	*P*	OR	95% CI
Gender			.605					
Male	309	84.4		Ref	–			
Female	57	15.6		1.187	0.619-2.279			
Age (years)			.872					
<60	322	88.0		Ref	–			
≥60	44	12.0		1.125	0.649-1.648			
Tumor max length (cm)			.001			.660		
<8	267	73.0		Ref	–			
≥8	99	27.0		1.660	1.354-2.614			
Tumor number			.442					
Single	275	75.1		Ref	–			
Multiple	91	24.9		1.261	0.698-2.278			
BCLC stage			.003			.555		
A	200	54.6		Ref	–			
B	166	45.4		2.103	1.287-3.437			
AFP (ng/mL)			.006			.029		
<400	177	48.3		Ref	–		Ref	–
≥400	189	51.7		2.008	1.220-3.311		2.079	1.076-4.016
Cirrhosis			<.001			.266		
No	283	77.3		Ref	–			
Yes	83	22.7		2.662	1.604-4.416			
Differentiation grade			<.001			.001		
Edmondson-Steiner I/II	224	61.2		Ref	–		Ref	–
Edmondson-Steiner III/IV	142	38.8		2.363	1.963-3.509		3.377	1.664-5.852
MVI			<.001			.008		
No	217	59.3		Ref	–		Ref	–
Yes	149	40.7		2.734	1.672-4.471		1.354	1.165-1.867
Satellite foci			<.001			<.001		
No	229	62.6		Ref	–		Ref	–
Yes	137	37.4		2.481	1.774-4.069		1.354	1.165-1.867
HBsAg			.057					
No	50	13.7		Ref	–			
Yes	316	86.3		1.551	0.941-2.164			
ALB			.847					
<35 g/L	166	45.4		Ref	–			
≥35 g/L	200	54.6		1.044	0.644-1.694			
ALT (U/L)			.240					
<100	291	79.5		Ref	–			
≥100	75	20.5		1.390	0.802-2.410			
AST (U/L)			.051					
<80	208	56.8		Ref	–			
≥80	158	43.2		1.637	0.988-2.660			
ALP (U/L)			.243					
<100	288	78.7		Ref	–			
≥100	78	21.3		1.395	0.797-2.440			
GGT (U/L)			.214					
<60	211	57.7		Ref	–			
≥60	155	42.3		2.027	0.843-2.781			
Timing of hepatectomy			<.001			<.001		
SDPH	241	65.8		Ref	–		Ref	–
SEPH	125	34.2		0.315	0.183-0.611		0.560	0.279-0.890
Hepatectomy time (minutes)			.715					
≥220	194	53.0		Ref	–			
<220	172	47.0		1.412	0.708-2.133			
Extent of hepatectomy			.078					
Major hepatectomy	188	51.4		Ref	–			
Minor hepatectomy	178	48.6		1.202	0.856-1.858			

Abbreviations: AFP, alpha-fetoprotein; ALBI, albumin-bilirubin grade; ALP, alkaline phosphatase; ALT, alanine aminotransferase; AST, aspartate aminotransferase; BCLC, Barcelona Clinic Liver Cancer; GGT, γ-glutamyl transpeptidase; HBsAg, hepatitis B surface antigen; HCC, hepatocellular carcinoma; MVI, microvascular invasion; OR, odds ratio; PM, Peritoneal implantation Metastasis; rHCC, ruptured hepatocellular carcinoma; SDPH, staged delayed partial hepatectomy; SEPH, staged early partial hepatectomy; TACE, transcatheter arterial chemoembolization.

### Evaluation indicators for each model

The best-performing model was the deep learning (DL) model, which achieved the highest precision (0.928), recall (0.939), accuracy (0.939), F1 score (0.917), and ROC-AUC (0.943) in the 10-fold training cohort. In the validation cohort, this model also performed well with a precision of 0.916, recall of 0.910, accuracy of 0.910, F1 score of 0.908, and ROC-AUC of 0.928. In the test cohort, this model also performed well with a precision of 0.884, recall of 0.887, accuracy of 0.887, F1 score of 0.899, and ROC-AUC of 0.892. On the other hand, the SVM model had the lowest performance. It obtained a precision of 0.734, recall of 0.728, accuracy of 0.728, F1 score of 0.745, and ROC-AUC of 0.733 in the 10-fold training cohort. In the validation cohort, the precision dropped to 0.699, recall to 0.682, accuracy to 0.682, F1 score to 0.715, and ROC-AUC to 0.671. In the test cohort, the precision dropped to 0.671, recall to 0.655, accuracy to 0.655, F1 score to 0.709, and ROC-AUC to 0.623 (Table 3). To assess the robustness of the model in a more homogeneous patient population, we conducted a subgroup analysis of patients undergoing staged early liver resection. We evaluated the model’s performance metrics in this subgroup to determine its predictive ability in the absence of variability in surgical timing. The model’s performance remained robust in the cohort of patients undergoing staged early liver resection. In the 10-fold training cohort, the ROC-AUC was 0.931, the accuracy was 0.924, the recall was 0.917, and the F1 score was 0.906. This indicates that even in the absence of variability in surgical timing, the model’s ability to predict postoperative PM remains quite strong (Table S4).

**Table 3.** Evaluation indicators for each model

Model	Precision	Recell	Accuracy	F1 score	ROC-AUC	95%CI
Logistic regression						
Average 10-fold training	0.876	0.883	0.883	0.881	0.915	0.816-0.983
Validation	0.866	0.871	0.871	0.869	0.908	0.820-0.989
Test	0.854	0.849	0.849	0.854	0.891	0.797-0.982
Classification tree						
Average 10-fold training	0.817	0.806	0.806	0.801	0.811	0.738-0.905
Validation	0.744	0.749	0.749	0.736	0.731	0.678-0.834
Test	0.729	0.730	0.730	0.728	0.723	0.661-0.828
Random forest						
Average 10-fold training	0.881	0.879	0.879	0.864	0.888	0.795-0.968
Validation	0.869	0.870	0.870	0.857	0.885	0.806-0.974
Test	0.852	0.834	0.834	0.546	0.847	0.784-0.935
Support vector machine						
Average 10-fold training	0.734	0.728	0.728	0.745	0.733	0.648-0.834
Validation	0.699	0.682	0.682	0.715	0.671	0.596-0.776
Test	0.671	0.655	0.655	0.709	0.623	0.553-0.704
Deep learning						
Average 10-fold training	0.928	0.939	0.939	0.917	0.943	0.874-0.992
Validation	0.916	0.910	0.910	0.908	0.928	0.845-0.971
Test	0.884	0.887	0.887	0.899	0.892	0.799-0.964

Abbreviations: AUC, area under the curve; ROC, receiver operator characteristic.

### PM patients’ characteristics and prognosis

The occurrence of peritoneal recurrence was observed in 78 patients. Table S5 describes the characteristics of these patients. A median AFP level of 116 (8-687) ng/mL was detected at the time of PM occurrence. Among them, 38 patients underwent peritoneal tumor resection, with a survival time of 28.2 ± 4.3 months, which was the longest prognosis among the 4 treatment methods (Table S5).

## Discussion

Malignant tumors of the abdominal organs often present a challenging issue of intra-abdominal implantation and metastasis post resection, particularly in cases of ruptured liver cancer. Previous researchers have reported varying rates of postoperative PM in different HCC patients, ranging from 7.7% to 40.7%, significantly higher than that observed in non-ruptured HCC patients.^7,14^ The covert and infiltrative growth of these tumors into other organs renders subsequent surgical intervention more difficult, and other treatment options are relatively suboptimal, severely impacting postoperative survival and quality of life. In our multicenter cohort of 522 patients with rHCC, a total of 78 patients developed postoperative PM. This subgroup of patients exhibited significantly poorer prognosis, with a 1-year OS rate of 51.3%. These findings are consistent with previous research, as demonstrated by Roussel et al^6^, whose results indicated that patients with postoperative PM had lower OS rates compared to rHCC patients with solitary tumor recurrence (21.53 months vs 9.76 months). Therefore, early identification of high-risk factors for postoperative intra-abdominal implantation in rHCC patients and the implementation of appropriate preventive and therapeutic measures are of utmost importance to reduce the incidence of postoperative PM.

In recent years, the adoption of machine learning analytics in clinical research has seen a significant rise. Leveraging artificial intelligence, these methods demand minimal human intervention and possess the capability to autonomously explore hyperparameters, effectively reducing biases and subjective errors commonly encountered in predictive models.^15,16^ Previous studies have not explored the prediction of postoperative PM in rHCC, making the current research novel and significant. We developed and validated traditional logistic regression models and 4 machine learning-based prediction tools using multicenter data. The DL model demonstrated the optimal discriminative power for predicting PM (10-fold training cohort AUC: 0.943; validation cohort ROC: 0.928; test cohort AUC: 0.892), while the RF model exhibited a comparable but suboptimal discriminative performance (10-fold training cohort AUC: 0.888; validation cohort: 0.885; test cohort AUC: 0.847), comparable to the traditional logistic regression model, which remained robust in its performance. The logistic models accurately predict postoperative PM and provide specific insights into the high-risk factors influencing PM. Although artificial intelligence allows the development of high-performance predictive models, the specific impact of each factor on postoperative PM may not be entirely clear, and the underlying relationships between these factors remain hidden. Taking this into consideration, we constructed decision trees with relatively shallow depths. From the trees, it becomes evident that tumor diameter, microvascular invasion (MVI), and timing of hepatectomy are the main branching points. Additionally, we calculated the feature importance in the RF model, which included the top 5 influential features, and represented them using a horizontal bar chart. The top 3 important features were tumor diameter, timing of hepatectomy, and AFP level. These findings align with the results of the traditional logistic regression model, wherein AFP, tumor diameter, MVI, and timing of hepatectomy were all statistically significant factors.

It is worth noting that the timing of hepatectomy is a significant predictor of postoperative PM. Previous studies at our center have suggested that patients who undergo SEPH have better prognoses compared to those who undergo SDPH.^13^ Therefore, we recommend performing staged early hepatectomy in resectable rHCC patients. Similarly, in the current study, SEPH was found to reduce the risk of postoperative PM (OR = 0.560 [0.279-0.890]), further reinforcing its importance and providing evidence in favor of staged early hepatectomy. However, there are some differences from previous research.^17-21^ For instance, Ren et al^22^ compared the incidence of PM between the emergency hepatectomy group and the delayed hepatectomy group (40.7% vs 35.3%) and found no statistically significant difference. They concluded that although patients in the staged group had lower disease-free survival rates after 6 months, there was no increase in recurrence rate or PM incidence. At the same time, previous related research has also suggested that both emergency hepatectomy and delayed hepatectomy do not exert a significant influence on postoperative PM.^14,23^ However, this may be attributed to the small sample size (*n* < 50). Additionally, the reliance on imaging-based diagnosis for postoperative PM, lack of pathological evidence, and variations in postoperative follow-up time may also influence these study outcomes. Previous researchers have emphasized that these conclusions need validation through large-scale, multicenter studies with long-term follow-up. Nevertheless, in our research with a substantial sample size and precise follow-up, we observed a lower rate of PM in patients who underwent staged early hepatectomy (11.1% vs 22.3%). Therefore, considering the comprehensive evidence, we continue to recommend staged early hepatectomy for patients. This approach appears to yield a lower risk of postoperative PM, which may enhance the overall prognosis and improve patient outcomes.

Our model suggests that risk factors for postoperative PM include tumor maximum diameter >8 cm, AFP levels ≥400 ng/mL, presence of MVI, and poorly differentiated tumors, which aligns with findings from previous literature.^24^ Portolani et al^24^ proposed a correlation between peritoneal implantation and aggressive tumor biology, with surgical treatment potentially achieving relatively acceptable survival rates. Regarding the prevention and treatment of postoperative peritoneal implantation, different researchers hold various perspectives. Huang et al^25^ found that a 15-minute lavage with sterile distilled water effectively kills tumor cells and significantly reduces the incidence of postoperative PM. This control technique can be considered for practical implementation during surgery. Baimas-George et al^26^ used laparoscopic microwave ablation and lavage of the peritoneal cavity in rHCC patients, achieving hemostasis and reducing the risk of peritoneal cancer, resulting in beneficial outcomes. Similarly, Kwak et al^27^ used intraoperative radiofrequency ablation combined with distilled water lavage to treat rHCC patients, markedly reducing the likelihood of postoperative peritoneal implantation.

Deep learning models have superior nonlinear modeling capabilities compared to multivariate logistic models, especially for complex clinical outcomes like postoperative PM, where multiple factors may interact in intricate ways. The DL models also exhibit higher AUC and F1 scores than logistic models. In practical clinical applications, clinical data can be integrated with such DL models into medical information systems. After surgery, the patient’s clinical and pathological data will be entered into the machine learning model to predict the risk of PM following the procedure. For patients predicted to be at high risk, the clinical team will take more intensive monitoring and treatment measures based on the model’s guidance. It is recommended that for high-risk patients, the frequency of imaging exams (such as contrast-enhanced CT or MRI) be increased, typically to follow ups every 3 months, compared to the standard follow-up frequency, which may be 6 months or longer. Early identification of metastatic lesions can significantly improve patient survival rates. High-risk patients may benefit from early chemotherapy or targeted therapy to reduce the spread of micrometastases or free cancer cells. Intraoperative peritoneal lavage may be used during the surgery to reduce the implantation of free cancer cells on intra-abdominal organs. Based on the prediction results of the machine learning model, physicians can fully discuss the postoperative treatment plan with the patient and their family, helping the patient understand their individualized postoperative risk and participate more actively in treatment decisions. This informed medical decision-making approach not only improves patient adherence but also enhances their quality of life.^28^ Subsequently, we further analyzed the model’s performance in the patient population that underwent only SEPH and compared it with the overall patient group. By quantifying performance differences, we identified that the model maintains high performance in this specific important cohort. Future research could optimize the model by collecting more data from patients treated solely with SEPH, enhancing its predictive ability in this subgroup.

Although our study represents the first large-sample prediction of postoperative PM in resectable HCC patients, there are still some limitations to consider. First, all our samples were derived from the Asian region, lacking validation from European samples. Notably, the main etiological factor for HCC in Asia is Hepatitis B virus (HBV) infection, which differs from the predominant factors in Western countries. Second, the level of evidence from our research remains weaker than prospective clinical trials. Future investigations should include more large-scale, multicenter clinical trials to strengthen the evidence base. Third, the depth of the DL model used in our study is relatively limited. Employing neural networks with more hidden layers may yield improved predictive results.

## Conclusion

In this large-scale multicenter Chinese cohort study aimed at establishing a postoperative PM prediction model, we observed that 14.9% of patients experienced PM. By utilizing machine learning methods, we developed accurate prognostic tools to predict individualized postoperative PM risk based on clinical and pathological characteristics. Among the 5 models constructed, the DL model exhibited the highest discriminative power and F1 score, indicating its superior performance in distinguishing and predicting PM outcomes.

## Supplementary Material

oyae341_suppl_Supplementary_Tables_S1

oyae341_suppl_Supplementary_Figures_S1

oyae341_suppl_Supplementary_Figures_S2

oyae341_suppl_Supplementary_Tables_S2

oyae341_suppl_Supplementary_Tables_S3

oyae341_suppl_Supplementary_Figures_S3

oyae341_suppl_Supplementary_Figures_S4

oyae341_suppl_Supplementary_Tables_S4

oyae341_suppl_Supplementary_Tables_S5

## Data Availability

The datasets generated and/or analyzed during the current study are not publicly available due [These data involve other studies by our team] but are available from the corresponding author on reasonable request.

## References

[1] Xia F , Ndhlovu E, Zhang M, et al Ruptured hepatocellular carcinoma: current status of research. Front Oncol. 2022;12:848903. https://doi.org/10.3389/fonc.2022.848903 35252016 PMC8891602

[2] Yoshida H , Mamada Y, Taniai N, Uchida E. Spontaneous ruptured hepatocellular carcinoma. Hepatol Res. 2016;46:13-21. https://doi.org/10.1111/hepr.12498 25631290

[3] Xia F , Zhang Q, Chen X, et al Comparison of the prognosis of BCLC stage A ruptured hepatocellular carcinoma patients after undergoing transarterial chemoembolization (TACE) or hepatectomy: a propensity score-matched landmark analysis. Surg Endosc. 2022;36:8992-9000. https://doi.org/10.1007/s00464-022-09351-2 35920912

[4] Xia F , Huang Z, Zhang Q, et al Hepatectomy for ruptured hepatocellular carcinoma classified as Barcelona Clinic Liver Cancer stage 0/A: the optimal treatment. Eur J Surg Oncol. 2022;48:2014-2022. https://doi.org/10.1016/j.ejso.2022.05.006 35595579

[5] Huang A , Guo DZ, Wang YP, et al The treatment strategy and outcome for spontaneously ruptured hepatocellular carcinoma: a single-center experience in 239 patients. J Cancer Res Clin Oncol. 2022;148:3203-3214. https://doi.org/10.1007/s00432-022-03916-3 35118561 PMC11800789

[6] Roussel E , Bubenheim M, Le Treut YP, et al; FRENCH Network. Peritoneal carcinomatosis risk and long-term survival following hepatectomy for spontaneous hepatocellular carcinoma rupture: results of a Multicenter French Study (FRENCH-AFC). Ann Surg Oncol. 2020;27:3383-3392. https://doi.org/10.1245/s10434-020-08442-5 32285281

[7] Kwak MS , Lee JH, Yoon JH, et al Risk factors, clinical features, and prognosis of the hepatocellular carcinoma with peritoneal metastasis. Dig Dis Sci. 2012;57:813-819. https://doi.org/10.1007/s10620-011-1995-1 22147252

[8] Moris D , Chakedis J, Sun SH, et al Management, outcomes, and prognostic factors of ruptured hepatocellular carcinoma: a systematic review. J Surg Oncol. 2018;117:341-353. https://doi.org/10.1002/jso.24869 29116644

[9] Tartaglia N , Di Lascia A, Cianci P, et al Hemoperitoneum caused by spontaneous rupture of hepatocellular carcinoma in noncirrhotic liver. a case report and systematic review. Open Med. 2020;15:739-744. https://doi.org/10.1515/med-2020-0202 PMC7712383 33336031

[10] Joshi RM , Telang B, Soni G, Khalife A. Overview of perspectives on cancer, newer therapies, and future directions. Oncol Transl Med. 2024;10:105-109.

[11] Chan WH , Hung CF, Pan KT, et al Impact of spontaneous tumor rupture on prognosis of patients with T4 hepatocellular carcinoma. J Surg Oncol. 2016;113:789-795. https://doi.org/10.1002/jso.24245 27062288 PMC5071691

[12] Sonoda T , Kanematsu T, Takenaka K, Sugimachi K. Ruptured hepatocellular carcinoma evokes risk of implanted metastases. J Surg Oncol. 1989;41:183-186. https://doi.org/10.1002/jso.2930410310 2545975

[13] Wu JJ , Zhu P, Zhang ZG, et al Spontaneous rupture of hepatocellular carcinoma: optimal timing of partial hepatectomy. Eur J Surg Oncol. 2019;45:1887-1894. https://doi.org/10.1016/j.ejso.2019.02.033 31405632

[14] Yang T , Sun YF, Zhang J, et al Partial hepatectomy for ruptured hepatocellular carcinoma. Br J Surg. 2013;100:1071-1079. https://doi.org/10.1002/bjs.9167 23754648

[15] Calderaro J , Seraphin TP, Luedde T, Simon TG. Artificial intelligence for the prevention and clinical management of hepatocellular carcinoma. J Hepatol. 2022;76:1348-1361. https://doi.org/10.1016/j.jhep.2022.01.014 35589255 PMC9126418

[16] Chaudhary K , Poirion OB, Lu L, Garmire LX. Deep learning-based multi-omics integration robustly predicts survival in liver cancer. Clin Cancer Res. 2018;24:1248-1259. https://doi.org/10.1158/1078-0432.CCR-17-0853 28982688 PMC6050171

[17] Kwon JH , Song GW, Hwang S, et al Surgical outcomes of spontaneously ruptured hepatocellular carcinoma. J Gastrointest Surg. 2021;25:941-953. https://doi.org/10.1007/s11605-020-04555-0 32246392

[18] Lee HS , Choi GH, Kang DR, et al Impact of spontaneous hepatocellular carcinoma rupture on recurrence pattern and long-term surgical outcomes after partial hepatectomy. World J Surg. 2014;38:2070-2078. https://doi.org/10.1007/s00268-014-2502-6 24663479

[19] Matsukuma S , Sato K. Peritoneal seeding of hepatocellular carcinoma: clinicopathological characteristics of 17 autopsy cases. Pathol Int. 2011;61:356-362. https://doi.org/10.1111/j.1440-1827.2011.02669.x 21615611

[20] Xu K , Ryu DH, Choi JW, et al Clinical impact of surgical treatment for the spontaneously ruptured resectable hepatocellular carcinoma: a single institution experience. Medicine (Baltimore). 2022;101:e30307. https://doi.org/10.1097/MD.0000000000030307 36107587 PMC9439726

[21] Zhang W , Huang Z, Che X. Emergency versus delayed hepatectomy following transarterial embolization in spontaneously ruptured hepatocellular carcinoma survivors: a systematic review and meta-analysis. World J Surg Oncol. 2022;20:365. https://doi.org/10.1186/s12957-022-02832-7 36397082 PMC9673318

[22] Ren A , Luo S, Ji L, et al Peritoneal metastasis after emergency hepatectomy and delayed hepatectomy for spontaneous rupture of hepatocellular carcinoma. Asian J Surg. 2019;42:464-469. https://doi.org/10.1016/j.asjsur.2018.09.006 30420157

[23] Shuto T , Hirohashi K, Kubo S, et al Delayed hepatic resection for ruptured hepatocellular carcinoma. Surgery. 1998;124:33-37. 9663249

[24] Portolani N , Baiocchi GL, Gheza F, et al Parietal and peritoneal localizations of hepatocellular carcinoma: is there a place for a curative surgery? World J Surg Oncol. 2014;12:298. https://doi.org/10.1186/1477-7819-12-298 25255984 PMC4190395

[25] Zhou SJ , Zhang EL, Liang BY, et al Distilled water lavage during surgery improves long-term outcomes of patients with ruptured hepatocellular carcinoma. J Gastrointest Surg. 2015;19:1262-1270. https://doi.org/10.1007/s11605-015-2797-0 25784370

[26] Baimas-George M , Watson M, Murphy KJ, et al Treatment of spontaneously ruptured hepatocellular carcinoma: use of laparoscopic microwave ablation and washout. HPB J. 2021;23:444-450. https://doi.org/10.1016/j.hpb.2020.08.001 32994101

[27] Kwak BJ , Park J, Kwon YK, Kwon JH, Yoon YC. Intraoperative radiofrequency ablation and distilled water peritoneal lavage for spontaneously ruptured hepatocellular carcinoma. Ann Surg Treat Res. 2019;97:291-295. https://doi.org/10.4174/astr.2019.97.6.291 31824883 PMC6893220

[28] Tran KA , Kondrashova O, Bradley A, et al Deep learning in cancer diagnosis, prognosis and treatment selection. Genome Med. 2021;13:152. https://doi.org/10.1186/s13073-021-00968-x 34579788 PMC8477474

