# Supplementary material for: Machine learning models for predicting postoperative peritoneal metastasis after hepatocellular carcinoma rupture: a multicenter cohort study in China
Source: Oncologist. 2025 Jan 20;30(1):oyae341. doi: 10.1093/oncolo/oyae341 (PMC11745018; doi:10.1093/oncolo/oyae341)
Supplement: oyae341_suppl_Supplementary_Tables_S1 [file oyae341_suppl_supplementary_tables_s1.docx]

Supplementary Table S1. Baseline characteristics of ruptured hepatocellular carcinoma patients with/without peritoneal implant metastasis undergoing hepatectomy in the whole cohorts before IPTW and after IPTW (n = 522).

|  |  | **Before-IPTW** | |  | **After-IPTW** | |  |
| --- | --- | --- | --- | --- | --- | --- | --- |
|  |  | **No-PM(n=444)** | **PM(n=78)** | **P-value**^‡^ | **No-PM** | **PM** | **P-value**^‡^ |
| Gender |  |  | | 0.011 |  | | 0.515 |
|  | Male | 380(85.6) | 65(83.3) |  | 805.7(89.5) | 435.8(84.9) |  |
|  | Female | 64(14.4) | 13(16.7) |  | 94.5(10.5) | 77.5(15.1) |  |
| Age |  |  |  | 0.589 |  |  | 0.390 |
|  | <60 y | 386(86.9) | 66(84.6) |  | 703.0(78.4) | 390.4(76.1) |  |
|  | ≥60 y | 58(13.1) | 12(15.4) |  | 197.1(21.6) | 122.9(23.9) |  |
| Tumor max length | |  |  | 0.068 |  |  | 0.053 |
|  | <8cm | 332(74.8) | 50(64.1) |  | 696.7(77.4) | 373.5(72.8) |  |
|  | ≥8cm | 112(25.2) | 28(35.9) |  | 203.4(22.6) | 139.8(27.2) |  |
| Tumor number | |  |  | 0.531 |  |  | 0.205 |
|  | Single | 335(75.5) | 62(79.5) |  | 711.0(81.0) | 390.4(76.1) |  |
|  | Multiple | 109(24.5) | 16(20.5) |  | 189.1(19.0) | 122.9(23.9) |  |
| BCLC stage |  |  |  | 0.004 |  |  | 0.679 |
|  | A | 258(58.1) | 31(39.7) |  | 611.8(68.0) | 343.1(66.8) |  |
|  | B | 186(41.9) | 47(60.3) |  | 288.3(32.0) | 170.2(33.2) |  |
| AFP |  |  |  | <0.001 |  |  | 0.204 |
|  | <400ng/ml | 270(60.8) | 29(37.2) |  | 161.3(17.9) | 105.9(20.6) |  |
|  | ≥400ng/ml | 174(39.2) | 49(62.8) |  | 738.8(82.1) | 407.4(79.4) |  |
| Cirrhosis |  |  |  | <0.001 |  |  | 0.285 |
|  | No | 352(79.3) | 46(59.0) |  | 790.6(87.8) | 440.8(85.9) |  |
|  | Yes | 92(20.7) | 32(41.0) |  | 109.5(12.2) | 72.5(14.1) |  |
| Differentiation grade | |  |  | <0.001 |  |  | 0.320 |
|  | Edmondson-Steiner I/II | 249 (56.1) | 15(19.2) |  | 706.0(78.4) | 390.2(76.0) |  |
|  | Edmondson-Steiner Ⅲ/Ⅳ | 195(43.9) | 63(80.8) |  | 194.1(21.6) | 123.1(24.0) |  |
| MVI |  |  |  | <0.001 |  |  | 0.178 |
|  | No | 291(65.5) | 32(41.0) |  | 706.1(78.5) | 316.6(61.7) |  |
|  | Yes | 153(34.5) | 46(59.0) |  | 194.0(21.5) | 196.7(38.3) |  |
| Satellite foci | |  |  | <0.001 |  |  | 0.101 |
|  | No | 302(68.0) | 27(34.6) |  | 527.7(58.6) | 324.3(63.2) |  |
|  | Yes | 142(32.0) | 51(65.4) |  | 372.4(41.4) | 189.0(36.8) |  |
| HBsAg |  |  |  | 0.001 |  |  | 0.207 |
|  | No | 70(15.8) | 1( 1.3) |  | 102.5( 11.4) | 70.9(13.8) |  |
|  | Yes | 374(84.2) | 77(98.7) |  | 797.6(88.6) | 442.4(86.2) |  |
| ALB |  |  |  | 0.958 |  |  | 0.184 |
|  | <35g/L | 204(45.9) | 35(44.9) |  | 454.8(52.7) | 240.4(46.8) |  |
|  | ≥35g/L | 240(54.1) | 43(55.1) |  | 445.3(47.3) | 272.9(53.2) |  |
| ALT |  |  |  | 0.303 |  |  | 0.899 |
|  | <100U/L | 351(79.1) | 57(73.1) |  | 678.6(75.4) | 400.1(78.0) |  |
|  | ≥100U/L | 93(20.9) | 21(26.9) |  | 221.5(24.6) | 113.2(22.0) |  |
| AST |  |  |  | 0.060 |  |  | 0.241 |
|  | <80U/L | 248(55.9) | 34(43.6) |  | 523.9(58.2) | 281.9(54.9) |  |
|  | ≥80U/L | 196(44.1) | 44(56.4) |  | 376.2(41.8) | 231.4(45.1) |  |
| ALP |  |  |  | 0.308 |  |  | 0.059 |
|  | <100U/L | 356(80.2) | 58(74.4) |  | 831.0(92.3) | 409.9(79.9) |  |
|  | ≥100U/L | 88(19.8) | 20(25.6) |  | 69.1(7.7) | 103.4(20.1) |  |
| GGT |  |  |  | 0.006 |  |  | 0.463 |
|  | <60U/L | 271(61.0) | 34(43.6) |  | 548.8(61.0) | 302.6(59.0) |  |
|  | ≥60U/L | 173(39.0) | 44(56.4) |  | 351.3(39.0) | 210.7(41.0) |  |
| Timing of hepatectomy | |  |  | <0.001 |  |  | 0.063 |
|  | SEPH | 305(68.7) | 38(48.7) |  | 639.0(71.0) | 339.5(66.1) |  |
|  | SDPH | 139(31.3) | 40(51.3) |  | 261.1(20.3) | 173.8(33.9) |  |
| Blood loss | | 348.61±50.59 | 343.58±48.46 | 0.415 | 348.95±49.98 | 348.95±49.98 |  |
| Perioperative blood transfusion |  |  |  | <0.001 |  |  | 0.210 |
|  | No | 305(68.7) | 9(11.5) |  | 692.6(76.9) | 314.2(61.2) |  |
|  | Yes | 139(31.3) | 69(88.5) |  | 207.5(23.1) | 199.1(38.8) |  |
| Times of HIO | |  |  | 0.001 |  |  | 0.265 |
|  | 0 | 168(60.0) | 51(39.8) |  | 513.8(57.1) | 277.1(54.0) |  |
|  | 1 | 84(30.0) | 58(45.3) |  | 386.3(42.9) | 236.2(46.0) |  |
| Time of inflow occlusion | | 13.18±4.31 | 11.07±3.96 | 0.013 | 12.99±4.14 | 13.26±4.28 | 0.244 |

The values in parentheses are percentages unless indicated otherwise.

^‡^ χ^2^ test with Yates’ correction.

Abbreviations: IPTW: Inverse Probability of Treatment Weighting; PM: Peritoneal Implant Metastasis; BCLC: Barcelona Clinic Liver Cancer; AFP: alpha-fetoprotein; HCC: hepatocellular carcinoma; rHCC: ruptured hepatocellular carcinoma; TACE: transcatheter arterial chemoembolization; MVI: microvascular invasion; HBsAg: hepatitis B surface antigen; ALBI: albumin–bilirubin grade; ALT: alanine aminotransferase; AST: aspartate aminotransferase; ALP: alkaline phosphatase; GGT: γ-glutamyl transpeptidase; SEPH: staged early partial hepatectomy; SDPH: staged delayed partial hepatectomy; HIO: hepatic inflow occlusion.
