# Supplementary figures and images for: Machine learning models for predicting postoperative peritoneal metastasis after hepatocellular carcinoma rupture: a multicenter cohort study in China
Source: Oncologist. 2025 Jan 20;30(1):oyae341. doi: 10.1093/oncolo/oyae341 (PMC11745018; doi:10.1093/oncolo/oyae341)

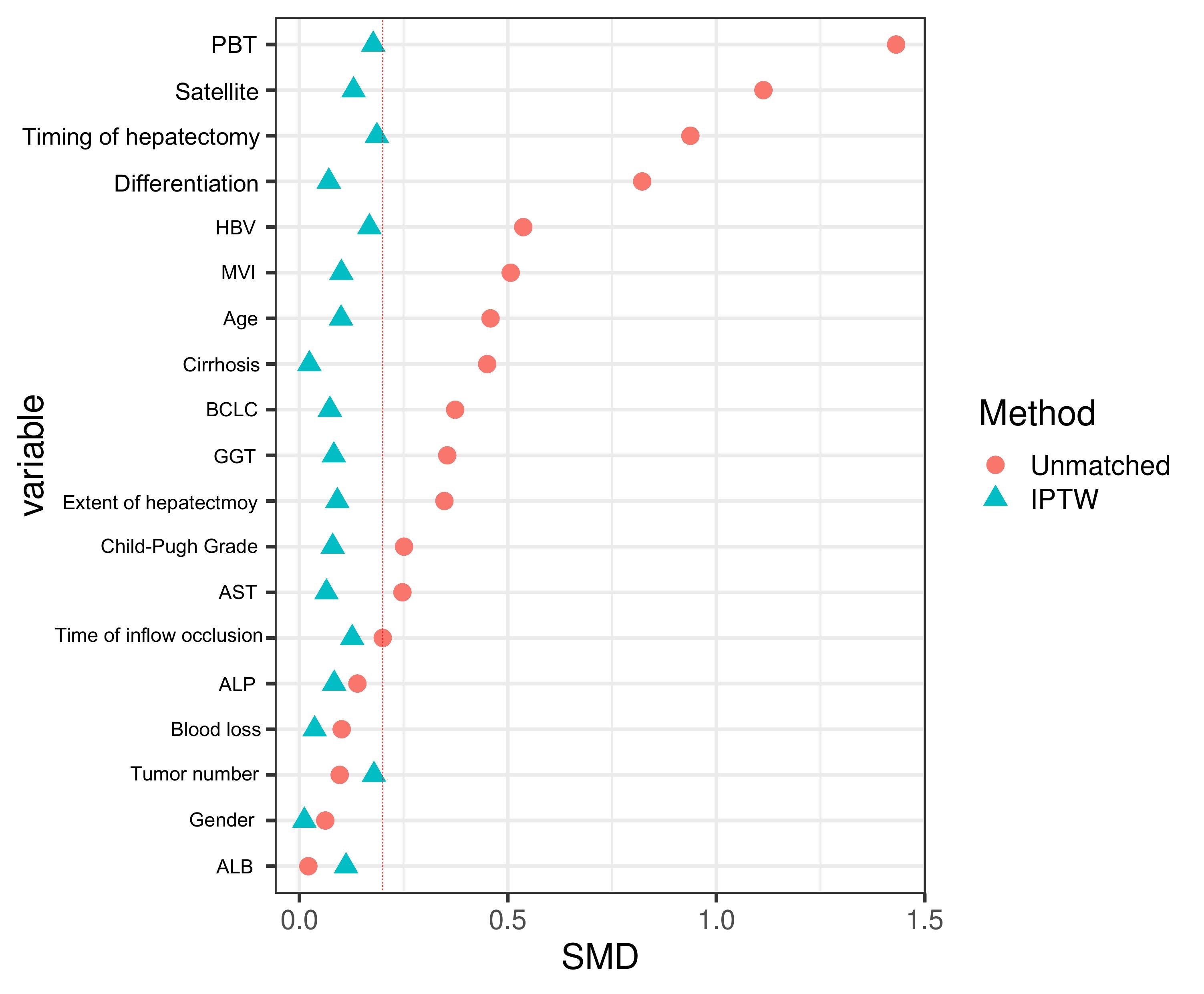

Supplement: oyae341_suppl_Supplementary_Figures_S1 [file oyae341_suppl_supplementary_figures_s1.jpeg]

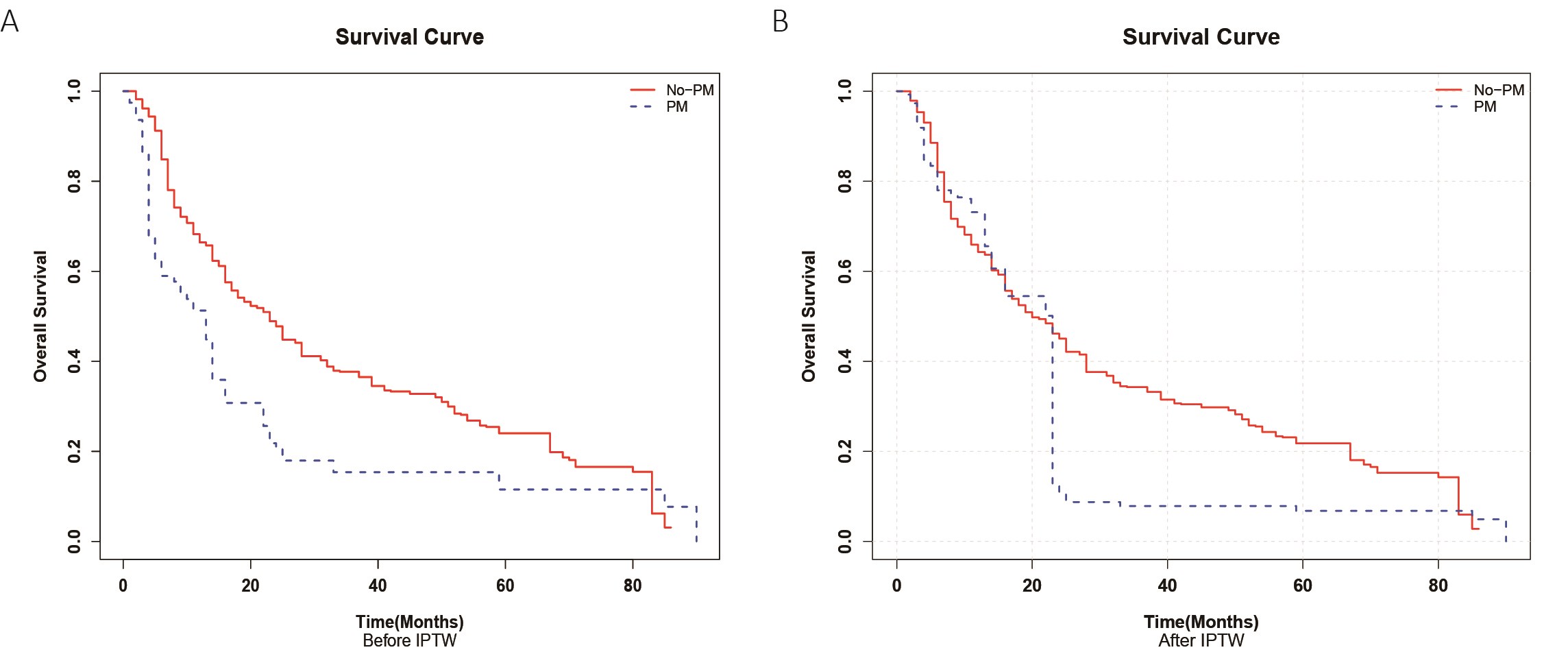

Supplement: oyae341_suppl_Supplementary_Figures_S2 [file oyae341_suppl_supplementary_figures_s2.jpeg]

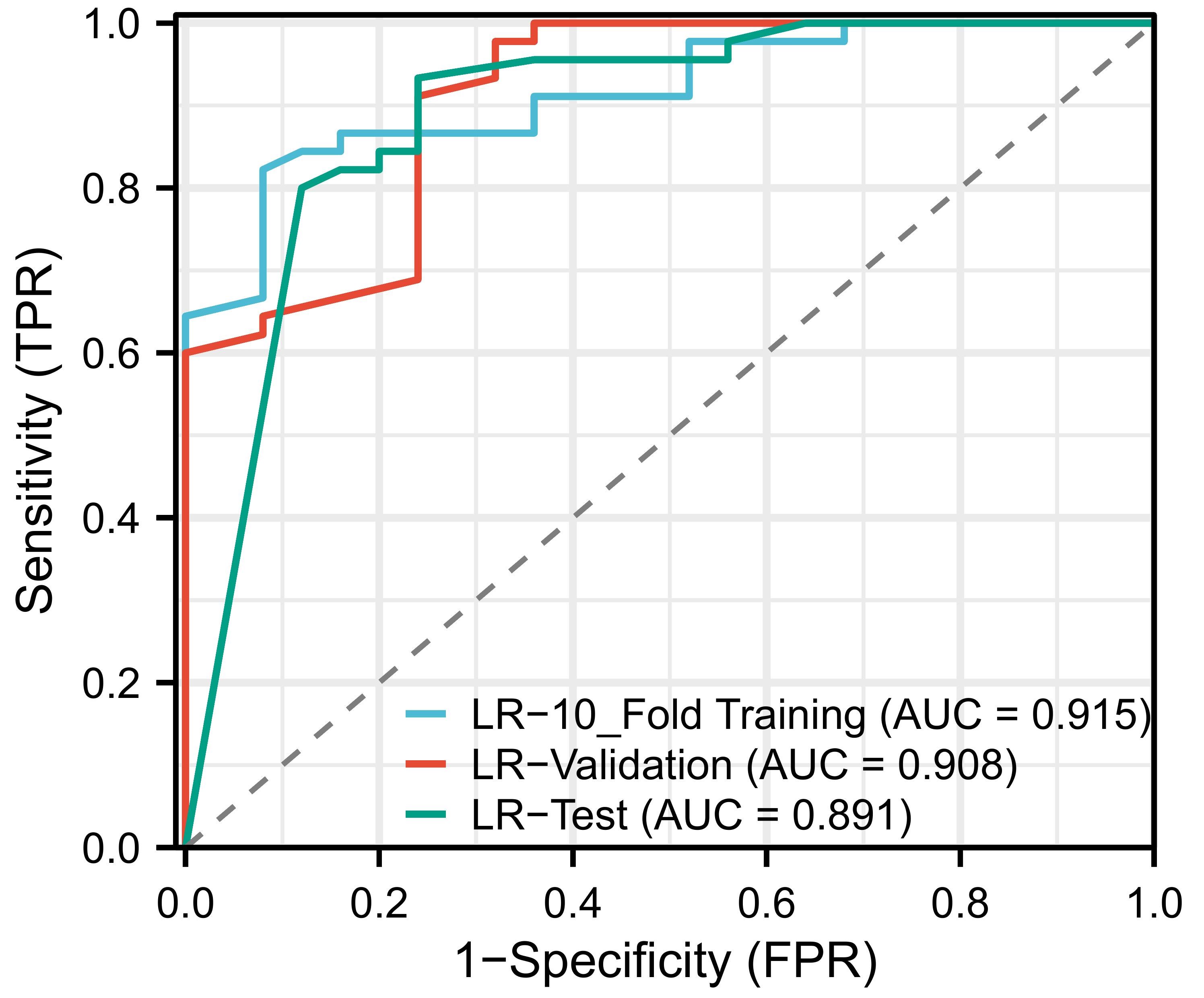

Supplement: oyae341_suppl_Supplementary_Figures_S3 [file oyae341_suppl_supplementary_figures_s3.jpeg]

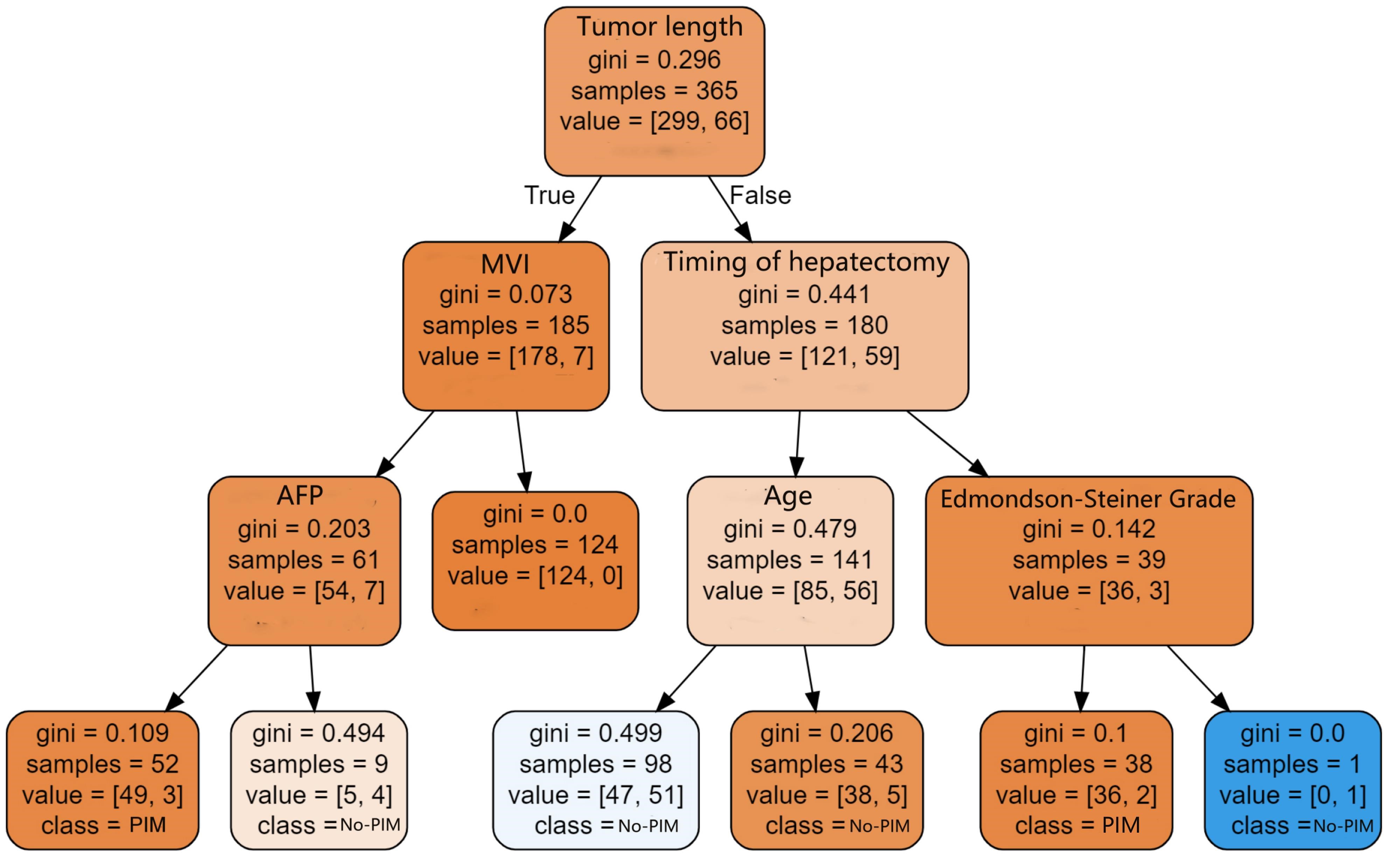

Supplement: oyae341_suppl_Supplementary_Figures_S4 [file oyae341_suppl_supplementary_figures_s4.jpeg]
