# Supplementary material for: Machine learning models for predicting postoperative peritoneal metastasis after hepatocellular carcinoma rupture: a multicenter cohort study in China
Source: Oncologist. 2025 Jan 20;30(1):oyae341. doi: 10.1093/oncolo/oyae341 (PMC11745018; doi:10.1093/oncolo/oyae341)
Supplement: oyae341_suppl_Supplementary_Tables_S2 [file oyae341_suppl_supplementary_tables_s2.docx]

Supplementary Table S2. Univariate and multivariate Cox proportional hazards regression analyses of predictors associated with overall survival (OS) in whole cohorts.

|  |  | Univariate Analysis | | |  | Multivariate Analysis | | |
| --- | --- | --- | --- | --- | --- | --- | --- | --- |
| Variables |  | P | HR | 95% CI |  | P | HR | 95% CI |
| Gender |  | 0.218 |  |  |  |  |  |  |
| Male |  |  | Ref | - |  |  |  |  |
| Female |  |  | 1.178 | 0.908-1.529 |  |  |  |  |
| Age (years) |  | **0.003** |  |  |  | 0.593 |  |  |
| <60 |  |  | Ref | - |  |  |  |  |
| ≥60 |  |  | 1.610 | 1.170-2.212 |  |  |  |  |
| Tumor max length(cm) |  | **0.007** |  |  |  | 0.018 |  |  |
| <8 |  |  | Ref | - |  |  | Ref | - |
| ≥8 |  |  | 1.372 | 1.088-1.731 |  |  | 1.391 | 1.088-1.731 |
| Tumor number |  | **<0.001** |  |  |  | 0.477 |  |  |
| Single |  |  | Ref | - |  |  |  |  |
| Multiple |  |  | 1.577 | 1.279-1.943 |  |  |  |  |
| BCLC stage |  | **<0.001** |  |  |  | <0.001 |  |  |
| A |  |  | Ref | - |  |  | Ref | - |
| B |  |  | 1.941 | 1.412-2.585 |  |  | 1.825 | 1.389-2.397 |
| AFP (ng/ml) |  | **<0.001** |  |  |  | <0.001 |  |  |
| <400 |  |  | Ref | - |  |  | Ref | - |
| ≥400 |  |  | 1.700 | 1.401-2.062 |  |  | 1.655 | 1.312-2.078 |
| Cirrhosis |  | **<0.001** |  |  |  | 0.288 |  |  |
| No |  |  | Ref | - |  |  |  |  |
| Yes |  |  | 1.438 | 1.155-1.792 |  |  |  |  |
| Differentiation grade |  | **<0.001** |  |  |  | <0.001 |  |  |
| Edmondson-Steiner I/II |  |  | Ref | - |  |  | Ref | - |
| Edmondson-Steiner Ⅲ/Ⅳ |  |  | 1.873 | 1.545-2.271 |  |  | 1.590 | 1.278-1.979 |
| MVI |  | **<0.001** |  |  |  | <0.001 |  |  |
| No |  |  | Ref | - |  |  | Ref | - |
| Yes |  |  | 2.341 | 1.920-2.855 |  |  | 1.701 | 1.304-2.219 |
| Satellite foci |  | **<0.001** |  |  |  | <0.001 |  |  |
| No |  |  | Ref | - |  |  | Ref | - |
| Yes |  |  | 2.086 | 1.711-2.542 |  |  | 2.259 | 1.788-2.852 |
| HBsAg |  | 0.267 |  |  |  |  |  |  |
| No |  |  | Ref | - |  |  |  |  |
| Yes |  |  | 1.185 | 0.879-1.597 |  |  |  |  |
| ALB |  | 0.594 |  |  |  |  |  |  |
| <35g/L |  |  | Ref | - |  |  |  |  |
| ≥35g/L |  |  | 0.949 | 0.783-1.150 |  |  |  |  |
| ALT(U/L) |  | 0.104 |  |  |  |  |  |  |
| <100 |  |  | Ref | - |  |  |  |  |
| ≥100 |  |  | 1.206 | 0.962-1.511 |  |  |  |  |
| AST(U/L) |  | **<0.001** |  |  |  | 0.005 |  |  |
| <80 |  |  | Ref | - |  |  | Ref | - |
| ≥80 |  |  | 1.734 | 1.429-2.104 |  |  | 1.466 | 1.121-1.916 |
| ALP(U/L) |  | **<0.001** |  |  |  | 0.002 |  |  |
| <100 |  |  | Ref | - |  |  | Ref | - |
| ≥100 |  |  | 2.154 | 1.707-2.719 |  |  | 1.670 | 1.207-2.310 |
| GGT(U/L) |  | **<0.001** |  |  |  | 0.031 |  |  |
| <60 |  |  | Ref | - |  |  | Ref | - |
| ≥60 |  |  | 1.718 | 1.414-2.087 |  |  | 1.351 | 1.028-1.776 |
| Timing of hepatectomy |  | **<0.001** |  |  |  | <0.001 |  |  |
| SDPH |  |  | Ref | - |  |  | Ref | - |
| SEPH |  |  | 2.484 | 2.031-3.037 |  |  | 2.081 | 1.681-2.713 |
| Hepatectomy time(min) |  | 0.338 |  |  |  |  |  |  |
| ≥220 |  |  | Ref | - |  |  |  |  |
| <220 |  |  | 1.178 | 0.875-1.825 |  |  |  |  |
| Extent of hepatectomy |  | **<0.001** |  |  |  | <0.001 |  |  |
| Major hepatectomy |  |  | Ref | - |  |  | Ref | - |
| Minor hepatectomy |  |  | 1.689 | 1.368-2.351 |  |  | 1.778 | 1.317-2.556 |
| Postoperative PM |  | **<0.001** |  |  |  | <0.001 |  |  |
| No |  |  | Ref | - |  |  | Ref | - |
| Yes |  |  | 1.871 | 1.450-2.414 |  |  | 1.388 | 1.179-1.988 |

Abbreviations: HR: hazards ratio; CI: confidence interval; BCLC :Barcelona Clinic Liver Cancer; AFP: alpha-fetoprotein; HCC: hepatocellular carcinoma; rHCC: ruptured hepatocellular carcinoma; TACE: transcatheter arterial chemoembolization; MVI: microvascular invasion; HBsAg: hepatitis B surface antigen; ALBI: albumin–bilirubin grade; ALT: alanine aminotransferase; AST: aspartate aminotransferase; ALP: alkaline phosphatase; GGT: γ-glutamyl transpeptidase; SEPH: staged early partial hepatectomy; SDPH: staged delayed partial hepatectomy; PM: Peritoneal Implant Metastasis
