# Supplementary material for: Machine learning models for predicting postoperative peritoneal metastasis after hepatocellular carcinoma rupture: a multicenter cohort study in China
Source: Oncologist. 2025 Jan 20;30(1):oyae341. doi: 10.1093/oncolo/oyae341 (PMC11745018; doi:10.1093/oncolo/oyae341)
Supplement: oyae341_suppl_Supplementary_Tables_S3 [file oyae341_suppl_supplementary_tables_s3.docx]

Supplementary Table S3. Short-term prognosis and postoperative complications in the PM group versus No-PM group.

|  | No-PM(n=444) | PM(n=78) | P-value§ |
| --- | --- | --- | --- |
| Number of deaths, n(%)† | 17(3.8) | 11(14.1) | 0.001 |
| Liver Failure, n(%) |  |  |  |
| Hemorrhagic complications leading to shock | 35(7.9) | 8(10.3) | 0.502 |
| Bleeding from ruptured esophageal varices | 62(14.0) | 12(15.4) | 0.725 |
| Hepatic encephalopathy | 27(6.1) | 5(6.4) | 0.803 |
| Massive ascites infection | 40(9.0) | 5(6.4) | 0.661 |
| Hydrocephalus, n(%) | 3(0.7) | 1(1.3) | 0.478 |
| Lung infection, n(%) | 9(2.0) | 2(2.6) | 0.673 |
| Distant metastasis of liver cancer‡, n(%) | 5(1.1) | 7(9.0) | <0.001 |
| Cardiovascular accidents, n(%) | 2(0.5) | 0(0.0) | 1.000 |
| Cerebrovascular accidents, n(%) | 4(0.9) | 1(1.3) | 0.556 |

† Patient died within three months after hepatectomy.

‡ indicates metastases to distant organs such as the heart, lungs, and gastrointestinal tract.

§Using the chi-square test or Fisher's exact probability test
