# Supplementary material for: Machine learning models for predicting postoperative peritoneal metastasis after hepatocellular carcinoma rupture: a multicenter cohort study in China
Source: Oncologist. 2025 Jan 20;30(1):oyae341. doi: 10.1093/oncolo/oyae341 (PMC11745018; doi:10.1093/oncolo/oyae341)
Supplement: oyae341_suppl_Supplementary_Tables_S4 [file oyae341_suppl_supplementary_tables_s4.docx]

Supplementary Table S4. Evaluation indicators for Deep Learning model in subgroup patients

| Model |  | Precision | Recell | Accuracy | F1 score | ROC-AUC | 95%CI |
| --- | --- | --- | --- | --- | --- | --- | --- |
| Deep Learning |  |  |  |  |  |  |  |
|  | Average 10-fold Training | 0.924 | 0.917 | 0.917 | 0.906 | 0.931 | 0.866-0.989 |
|  | Validation | 0.908 | 0.909 | 0.909 | 0.901 | 0.925 | 0.854-0.974 |
|  | Test | 0.876 | 0.862 | 0.862 | 0.887 | 0.901 | 0.821-0.955 |

Abbreviation: ROC: receiver operator characteristic; AUC: area under the curve; CI: confidence interval.
