# Supplementary material for: Machine learning models for predicting postoperative peritoneal metastasis after hepatocellular carcinoma rupture: a multicenter cohort study in China
Source: Oncologist. 2025 Jan 20;30(1):oyae341. doi: 10.1093/oncolo/oyae341 (PMC11745018; doi:10.1093/oncolo/oyae341)
Supplement: oyae341_suppl_Supplementary_Tables_S5 [file oyae341_suppl_supplementary_tables_s5.docx]

Supplementary Table S5. Clinical management and prognosis of all patients who developed peritoneal metastases.

|  | PM (n=78) |
| --- | --- |
| Number of deaths, n(%)† | 72(92.3) |
| Recurrence management, n(%) |  |
| Surgery | 38(48.7) |
| chemotherapy | 27(34.6) |
| sterile distilled water | 10(12.8) |
| best supportive care | 3(3.8) |
| OS§, x̄ ± s month |  |
| Surgery | 28.2±4.3 |
| chemotherapy | 22.0±4.5 |
| sterile distilled water | 14.1±4.4 |
| best supportive care | 13.8±6.3 |
| AFP‡ Median [25%-75%] | 116 [8-687] ng/mL |

† As of the latest follow-up date

‡ To monitor AFP at the time of peritoneal recurrence

§The prognosis resulting from the four treatment methods has a P-value <0.001

Abbreviation: OS:Overall Survival, AFP: Alpha-Fetoprotein
